# A male-killing *Wolbachia* carries a feminizing factor and is associated with degradation of the sex-determining system of its host

**DOI:** 10.1098/rsbl.2011.1114

**Published:** 2012-01-04

**Authors:** Takafumi N. Sugimoto, Yukio Ishikawa

**Affiliations:** Graduate School of Agricultural and Life Sciences, Department of Agriculture and Environmental Biology, The University of Tokyo, Tokyo 113-8657, Japan

**Keywords:** *Ostrinia scapulalis*, male killing, feminization, masculinization, *doublesex*, *Wolbachia*

## Abstract

Endosymbiotic bacteria of the genus *Wolbachia* induce diverse reproductive alterations in their insect hosts. *Wolbachia* (wSca) infecting the moth *Ostrinia scapulalis* causes unusual male killing, in which males (genotype: ZZ) selectively die during embryonic and larval development, whereas females (genotype: ZW), in turn, selectively die when cured of infection. To gain insight into the interaction between wSca and the host, we analysed phenotypic and genetic sexes of the embryos and larvae of normal, wSca-infected, and infected-and-cured *O. scapulalis* by diagnosing the sex-specifically spliced transcripts of *Osdsx*—a homologue of the sex-determining gene *doublesex*—and sex chromatin in interphase nuclei, respectively. It was observed that the female-type *Osdsx* was expressed in the infected male (ZZ) progenies destined to die, whereas the male-type *Osdsx* was expressed in the cured female (ZW) progenies destined to die. These findings suggest that (i) wSca, a male killer, carries a genetic factor that feminizes the male host, (ii) the sex-determining system of the host is degraded, and (iii) a mismatch between the genetic and phenotypic sexes underlies the sex-specific death.

## 1. Introduction

*Wolbachia*, a group of endosymbiotic bacteria harboured by a wide range of insects [1], is known for various manipulations of host reproduction to expedite their own propagation [2]. Among the manipulations is male killing, in which males selectively die during embryonic and larval development, giving rise to all-female progeny [3]. Despite the conspicuous effect of the infection, the molecular interactions between *Wolbachia* and its hosts that may mediate male-specific death have remained unexplored. *Wolbachia* (wSca) infecting the adzuki bean borer moth (*Ostrinia scapulalis*) causes male killing, however, the male killing in this *Wolbachia*–host system is unusual in that females, in turn, selectively died when wSca was eliminated by antibiotic treatment, giving rise to all-male progeny [4]. The sex dependence of death in this system suggests that wSca interferes with the sex-specific gene expression or physiology of the host. Indeed, the occurrence of sexual mosaic individuals with an exclusively male genotype upon incomplete elimination of wSca with antibiotics or heat treatment strongly suggests that wSca has the ability to feminize genetic males [4–6]. Until now, however, molecular interactions between the symbiont and host have not been investigated in this unique system.

In addition to *Wolbachia*, diverse bacteria from the genera such as *Spiroplasma*, *Rickettsia* and *Arsenophonus* are known to cause male-specific death in their hosts [3]. Although the mechanism of male killing has been studied in detail only in a few bacterium–host systems [7–9], these studies suggested that mechanisms of male killing might be diverse: dosage compensation is suggested to be involved in *Spiroplasma*-induced male killing in *Drosophila* [7], whereas *Arsenophonus* is reported to target maternally inherited centrosomes to kill males in *Nasonia* [9].

In the present study, to gain insight into the mechanism of male killing by *Wolbachia*, we focused on a gene working at the bottom of the sex-determination cascade, *doublesex* (*dsx*), which is transcribed into either a male or female isoform by sex-specific splicing and serves as a final regulator of sex-specific gene expression in somatic cells of insects [10,11]. We investigated the developmental changes in phenotypic and genetic sex ratios in the broods of normal, wSca-infected and infected-and-cured *O. scapulalis* by diagnosing the isoforms of a *dsx* homologue (female-specific *Osdsx*^*FL*^ or male-specific *Osdsx*^*M*^ [12]), and sex chromatin in interphase nuclei, respectively.

## 2. Material and methods

### (a) Insects

Adult moths of *O. scapulalis* (Lepidoptera: Crambidae) were captured at Matsudo, Japan (35.8° N, 139.9° E) in the summer of 2008–2009. Females infected with *Wolbachia*, which produce all-female offspring, were screened by diagnostic polymerase chain reaction (PCR; see §2*b*), and maintained as matrilines through crosses with uninfected normal males [4]. Three *Wolbachia*-infected matrilines and three uninfected cultures of *O. scapulalis* were used for the present study. Insects were reared at 23 ± 1°C with a photoperiod of 16 L : 8 D. The larvae were reared on an artificial diet (Silkmate 2M, Nosan Corp.) [4].

### (b) Diagnostic PCR

DNA was extracted from the ovaries of female moths using a DNeasy Tissue Kit (Qiagen), and PCR was performed using Ex Taq DNA polymerase (Takara Bio Inc.) and *wsp* gene-specific primers, *wsp*-81F and *wsp*-691R [13]. The primers used for amplification of the actin gene, as a positive control, were actin-F and actin-R [12].

### (c) Tetracycline treatment

*Wolbachia* was eliminated from the infected matrilines by rearing larvae on an artificial diet containing tetracycline hydrochloride (0.06%, w/w) throughout the entire larval stage. Only female adults were obtained in the generation treated. The absence of *Wolbachia* in the females was confirmed by diagnostic PCR. These *Wolbachia*-eliminated females, when crossed with normal males, produce all-male progeny [4,12].

### (d) Observation of the sex chromatin for genetic sexing

The genetic sex of individuals can be determined from the presence (female) or absence (male) of sex chromatin in interphase nuclei in the cells of Malpighian tubules or silk glands [4,12]. Malpighian tubules were dissected out from larvae or adults in sterile saline, and fixed with methanol : acetic acid (3 : 1) for approximately 1 min. The preparations were stained with lactic acetic orcein, and examined under a light microscope. In the case of embryos, embryos (pre-hatched larvae) were taken out from the eggs, and tissues including the Malpighian tubules and silk glands were isolated by pulling the abdominal tip with fine forceps. The remainder of the embryo was used for RNA extraction.

### (e) Analysis of the type of *Osdsx* expressed in embryos

Total RNA was isolated from embryos, larvae and adults of *Wolbachia*-infected, infected-and-cured and uninfected *O. scapulalis* using RNAiso (Takara Bio Inc.), and treated with RNase-free DNase I (Qiagen). First-strand cDNA was synthesized from the total RNA using a PrimeScript first-strand cDNA Synthesis Kit (Takara Bio Inc.). PCR amplification was performed using KOD FX DNA polymerase (Toyobo) with the primers exon 1-F and exon 5-R under the following conditions: 98°C for 2 min, 30 cycles of 98°C for 15 s, 60°C for 10 s, 68°C for 60 s and a final extension at 72°C for 10 min [12]. The sizes of the PCR products with the above primers were 468 bp for *Osdsx*^*M*^ and 725 bp for *Osdsx*^*FL*^.

## 3. Results

**Figure 1.**
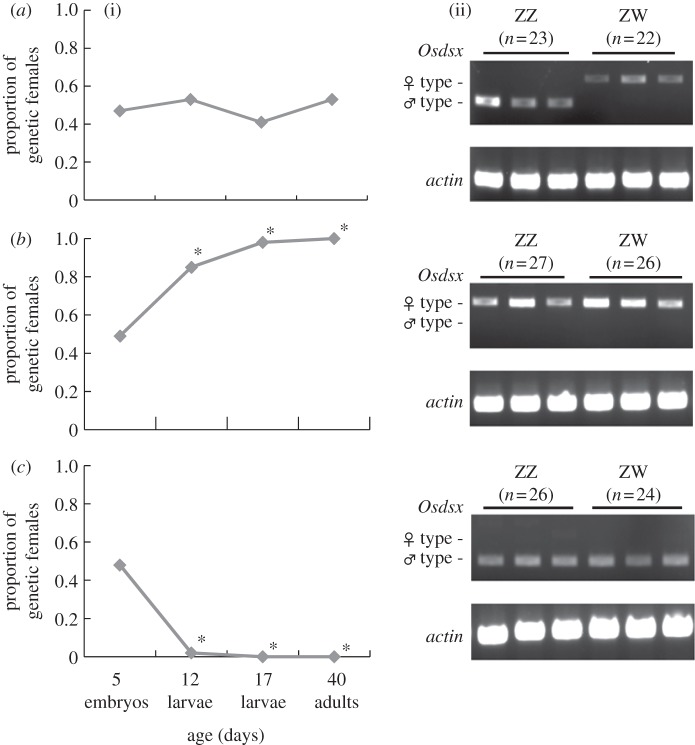
Changes in the genetic sex ratio associated with the development of *Ostrinia scapulalis* ((i) *n* = 23–92), and the expression of male and female-type *Osdsx* transcripts in 5-day-old embryos ((ii) only examples are shown). Age refers to days after oviposition. Genetic sex was determined by the presence/absence of sex chromatin in the cells [4]. The embryos (pre-hatched larvae) were checked for sex chromatin and the type of *Osdsx* (*Osdsx*^*M*^ or *Osdsx*^*FL*^, see the text). The actin gene was used as a reference. ZZ, male genotype; ZW, female genotype. Asterisks represent significantly different from 0.5 by Fisher's exact test (*p* < 0.01). *Wolbachia*: (*a*) uninfected; (*b*) infected and (*c*) eliminated.

In an uninfected normal strain of *O. scapulalis*, the sex ratio of individuals did not deviate significantly from 1 : 1 (proportion of females = 0.5) throughout development (figure 1*a*), and the type of *dsx* homologue expressed was in accordance with the genetic sex, that is, the male-type *Osdsx*^*M*^ was expressed in individuals with the ZZ genotype, and the female-type *Osdsx*^*FL*^ was expressed in individuals with the ZW genotype (figure 1*a*). We confirmed that all genetic males in the wSca-infected strain eventually died during the larval stage (figure 1*b*), and conversely, all genetic females freed from infection died at the early larval stage (figure 1*c*). Diagnoses of the *Osdsx* isoforms in the same individuals showed that the female-type *Osdsx*^*FL*^ was expressed in all individuals infected with wSca irrespective of genetic sex (ZZ and ZW; figure 1*b*), indicating that wSca feminized genetic males (ZZ). By contrast, the male-type *Osdsx*^*M*^ was expressed in all individuals freed from infection irrespective of the genetic sex (figure 1*c*), indicating that elimination of wSca brought about the masculinization of genetic females (ZW). Inviability of embryos/larvae was observed when the genetic sex and phenotypic sex differed (table 1).

**Table 1.** Viability of progenies produced by normal (uninfected), *Wolbachia*-infected and infected-and-cured *Ostrinia scapulalis* female moths with reference to the genotypic and phenotypic sexes of progenies. (W* indicates a W chromosome suggested to have a dysfunctional female-determining factor.)

infection state of mother	*Wolbachia*^a^	genotype^b^	sexual phenotype^c^	viability
uninfected	−	ZW	female	viable
		ZZ	male	viable
infected	+	ZW*	female	viable
		ZZ	female	inviable
cured^d^	−	ZW*	male	inviable
		ZZ	male	viable

^a^Minus and plus indicate absence and presence of *Wolbachia* in progenies, respectively.

^b^The genotype of an individual was determined by the presence/absence of sex chromatin in interphase nuclei, which is a condensed heterochromatin formed from the W chromosome.

^c^The sexual phenotype of an individual was determined by the sex-specific isoforms of *Osdsx*, a homologue of the sex-determining gene *doublesex* (see the text for more details).

^d^Cured of *Wolbachia* infection by tetracycline treatment.

## 4. Discussion

In the silkmoth (*Bombyx mori*), the presence of a single W chromosome ensures female development, whereas in its absence male development takes place [14]. Thus, the W chromosome is believed to carry an epistatic female-determining factor (referred to hereafter as the F factor), although its molecular nature as well as the transcriptional cascade leading to the female-specific splicing of *dsx* is largely unknown (reviewed in earlier studies [11,15,16]).

The expression of the female-type *Osdsx*^*FL*^ in genetic males (ZZ) of the wSca-infected *O. scapulalis* clearly indicates that wSca, a male killer, carries a feminizing factor that interferes with upstream sex-determination processes, or possibly the sex-specific splicing of *Osdsx* itself. Meanwhile, the masculinization of genetic females (ZW) freed from infection indicates that a factor in the female-determining cascade is degraded in the wSca-infected strains. Here, it should be noted that wSca-infected strains, which comprise females only, have been maintained by crossing with males from normal strains. Given that the degraded factor has been stably transmitted from mother to daughter, it is most likely to be located on the recombinationally isolated W chromosome, and it is possible that the F factor itself is degraded.

Although discordance of the genetic and phenotypic sexes is suggested to underlie the sex-specific death (table 1), the mechanism of death is unresolved. In the male killing, caused by wSca, (i) the growth of embryo/larvae destined to die was generally retarded, (ii) no discernible abnormalities were found in their morphology, and (iii) the occurrence of death was irregular. Therefore, the mechanism of death is not likely to be linked to a specific developmental process or event. One plausible explanation for growth retardation and eventual death may be discordance in dosage compensation, i.e. sex-dependent adjustment of the expression levels of genes on the sex chromosomes. Indeed, involvement of dosage compensation is suggested in the *Spiroplasma*-induced male killing in *Drosophila* [7]. In moths, however, the presence of dosage compensation is itself currently under discussion [17,18]. Studies on the expression levels of Z-linked genes in wSca-infected and infected-and-cured individuals may shed light on the dosage compensation in moths.

Our recent identification of sex-specific isoforms of *Osdsx* [12] paved the way for the simultaneous analysis of genetic and phenotypic sexes. It was hitherto difficult to determine the phenotypic sex of embryos and young larvae, because they do not show any sexual difference in morphology. Discordance between the genetic and phenotypic sexes in embryos/larvae destined to die was uncovered for the first time, to our knowledge, by this simultaneous analysis.

In addition to male killing, reproductive alterations performed by *Wolbachia* include, among others, feminization [2]. It is intriguing that a feminizing effect underlies the male killing, because feminized individuals are viable when produced by a ‘true’ feminizer in the butterfly, *Eurema hecabe* [19]. Comparison of the male killer in *O. scapulalis* and the feminizer in *E. hecabe* might shed light on how *Wolbachia* developed their repertoire of reproductive manipulations.
